# A comprehensive immune repertoire signature distinguishes pulmonary infiltration in SARS-CoV-2 Omicron variant infection

**DOI:** 10.3389/fimmu.2024.1486352

**Published:** 2024-12-17

**Authors:** Xuechuan Li, Hongyi Zhu, Peipei Xu, Jie Zhang, Zhe Wang, Hui He, Fang Shen, Yi Jiang, Lijuan Shen, Jing Xiang, Linhua Yang, Chao Yang, Hao Jiang, Ganglong Gao, Junshuo Jin, Huojian Shen, Yinping Wang, Linshi Wu, Changlin Qian, Dejun Liu, Weiqing Qiu, Qiwei Li, Yuanwen Chen, Fujun Lin, Yun Liu

**Affiliations:** ^1^ Department of Biliary-Pancreatic Surgery, Renji Hospital Affiliated to Shanghai Jiao Tong University, School of Medicine, Shanghai, China; ^2^ Tilcure Biotherapeutics, Shanghai, China; ^3^ Department of Training Department, China Medical University Benxi Central Hospital Postgraduate Training Workstation, Shanghai, China; ^4^ Renal Division, Department of Internal Medicine, Xinhua Hospital Affiliated to Shanghai Jiao Tong University School of Medicine, Shanghai, China; ^5^ Shanghai Cancer Institute, Shanghai, China

**Keywords:** COVID-19, SARS-CoV-2 Omicron variant, immune repertoire, vaccine, TCR

## Abstract

**Introduction:**

The coronavirus disease 2019 (COVID-19) global pandemic has been the most severe public health emergency since 2019. Currently, the Omicron variant of severe acute respiratory syndrome coronavirus 2 (SARS-CoV-2) has been the most dominant. The most prominent symptom of SARS-CoV-2 infection is respiratory. Meanwhile, the fatality of COVID-19 was mainly from pneumonia. However ,in patients with SARS-CoV-2 infection who have pneumonia and those who do not, the differences in the immune repertoire still require further investigation.

**Methods:**

We conducted seven-chain adaptome immune repertoire analyses on patients with SARS-CoV-2 Omicron infection, both with and without pulmonary infiltration.

**Results:**

Patients with pulmonary infiltration exhibit lymphopenia, a decreased proportion of the overall TCR repertoire alongside an increased BCR repertoire, reduced IGHD and IGHM isotype expression, a shorter mean CDR3 length for TRG, and a longer mean length for TRD, as well as diminished clonality and diversity in the TCR/BCR repertoire. Meanwhile, patients with pulmonary infiltration have distinct V-J gene usage and unique CDR3 signature, as well as BCR class switch recombination pattern. Finally, prior vaccination triggered less BCR IGHM/IGHD somatic hypermutation response, preserved the diversity of the entire adaptive immune repertoire, and provided clinical protection against severe or critical conditions following Omicron infection.

**Discussion:**

We report a unique, comprehensive adaptive immune system signature in patients with pulmonary infiltration, which may serve as potential immunological biomarkers and therapeutic targets.

## 1 Introduction

Coronavirus disease 2019 (COVID-19), caused by severe acute respiratory syndrome coronavirus 2 (SARS-CoV-2), had been posing a serious threat to global health (1). As of November 2022, SARS-CoV-2 had infected more than 600 million people and caused more than 6 million deaths worldwide (https://covid19.who.int/). Over the last 5 years of the COVID-19 pandemic, the SARS-CoV-2 virus had undergone a high frequency of mutations and had been evolving swiftly (41). Variants of concern have appeared at regular intervals—Alpha, Beta, Gamma, Delta, and now Omicron. The Omicron variant, first identified in Botswana in November 2021, was rapidly becoming the dominant circulating variant (1). The city of Shanghai, China, encountered a wave of SARS-CoV-2 Omicron spread since March 2022, which caused hundreds of casualties (2).

Although most patients with COVID-19 present with mild illness, even asymptomatic, some patients do develop severe pulmonary, which is an important risk factor for mortality (3–6). The Omicron variant exhibits a higher transmissibility than prior SARS-CoV-2 variants as well as the capability to evade naturally acquired and vaccine-induced immunity (7). As with any other virus infection, the adaptive immune response plays a central role in clearing SARS-CoV-2 (8, 9). In addition to providing host protection, adaptive immune functions may contribute pathologic mediators, including B-cell autoreactivities associated with specific disease-related characteristics in many patients with COVID-19 (10, 11). Severe disease and death caused by SARS-CoV-2 infection appear to be largely due to failures and/or dysregulation of the immune response in vulnerable populations. Currently, there is limited information on the impact of SARS-CoV-2 Omicron variant infection on the adaptive immune responses, especially in patients with pulmonary infiltration.

The immune system comprises innate immunity and adaptive immunity that offer protection against viruses and other pathogens. T cells and B cells are the central mediators of antiviral adaptive immunity (12). Of each T or B cell, there are unique T-cell receptors (TCRs) or B-cell receptors (BCRs), which are expressed on the cell surface. The antigen specificity of each TCR and BCR is primarily determined by the hypervariable complementarity-determining region 3 (CDR3) of the receptor chain, which originates from the recombination of the V (variable), D (diversity), and J (joining) gene segments and the deletion and insertion of nucleotides at the V(D)J junctions (13). Using immune repertoire next-generation sequencing, which offers in-depth quantitative and molecular-level profiling of immune repertoire, helps us understand the dynamics of the antigen-adaptive immune response in humans (14–16).

In humoral immunity, an antibody or immunoglobulin (Ig) can recognize a specific antigen through its N-terminal variable region and activate downstream immune effects through its C-terminal constant region. Upon antigen stimulation, B cells can further diversify the antibody gene by introducing mutations at the Ig variable region exon by somatic hypermutation (SHM) to allow antibody affinity maturation and by switching the antibody class through class switch recombination (CSR) to change the downstream effector functions (17, 18). Various studies have characterized the BCR repertoire feature for SARS-CoV-2 infection and vaccination, thus directing the therapeutic development as well as deepening our understanding of the immunological changes for SARS-CoV-2 infection (19, 20).

An efficacious vaccine is considered essential to prevent further morbidity and mortality from SARS-CoV-2 infection (21). The inactivated SARS-CoV-2 vaccines have shown effective immune responses in eliciting neutralizing antibodies in multiple clinical trials (22, 23). However, with the novel SARS-CoV-2 variants emerging and circulating widely, whether the original vaccines that were designed based on the wild-type SARS-CoV-2 can offer enough protection against emerging variants and the immunological reactions triggered by the vaccines has been under contentious debate (24).

Here, we investigated the peripheral blood immune repertoire of patients infected with the SARS-CoV-2 Omicron variant across different levels of clinical severity. Our goal was to identify a unique immune repertoire signature that could influence the determination of clinical outcomes for SARS-CoV-2 Omicron variant infection.

## 2 Methods

### 2.1 Enrollment of patients

The study protocol was approved by the Ethics Committee of Renji Hospital affiliated with Shanghai Jiao Tong University (KY2022-103-B). A total of 39 patients diagnosed with SARS-CoV-2 Omicron variant infection were enrolled. The classification was performed according to the Diagnosis and Treatment Plan for COVID-19 (trial version 9) recommended by the National Health Commission of the People’s Republic of China as follows: (I) mild type: clinical symptoms are mild, and there are no radiographic signs of pneumonia; (II) common type: the aforementioned clinical symptoms are exhibited, and radiographic imaging shows signs of pneumonia; and (III) severe type: adults meet any of the following criteria: shortness of breath with a respiratory rate (RR) ≥30 breaths/min, oxygen saturation (SpO_2_) ≤93% while breathing ambient air at rest, and arterial oxygen partial pressure (PaO_2_)/fraction of inspired oxygen (FiO_2_) ≤300 mmHg (1 mmHg = 0.133 kPa). In high-altitude areas (altitude over 1,000 m), PaO_2_/FiO_2_ should be adjusted using the following formula: PaO_2_/FiO_2_ × [760/atmospheric pressure (mmHg)]. In the severe type, there is also progressive worsening of clinical symptoms, with significant lesion progression >50% within 24–48 h as shown by radiographic imaging. (I) pertains to the mild disease group without pulmonary infiltration, and (II) and (III) refer to the severe/critical group with pulmonary infiltration. Data on age, sex, comorbidities, SARS-CoV-2 vaccination status, baseline value of cycle threshold, lymphocyte count, and prognosis were collected.

### 2.2 RNA extraction

Fresh blood samples (2 ml) were collected from the patients with informed consent, drawn into EDTA tubes, transferred immediately into 15 ml microcentrifuge tubes, and added with 10 ml of TRIzol LS reagent. Total RNAs were extracted according to the manufacturer’s protocol.

### 2.3 TCR and BCR immune repertoire sequencing

For the current study, we used commercially available iR-RepSeq-plus 7-Chain Cassette (iRepertoire, Inc., USA) to generate NGS libraries covering all TCR and BCR seven chains, namely, TCR alpha (TRA), beta (TRB), delta (TRD), and gamma (TRG) and BCR Ig heavy (IGH), Ig kappa (IGK), and Ig lambda (IGL) from the RNA template. All seven chains were amplified in a single assay using a strategy that allows the incorporation of unique molecular identifiers (UMIs) during the reverse transcription (RT) step. Each disposable cassette is for library preparation for one sample, and all necessary reagents for amplification and purification are preloaded into the cassette. The library size is 500 bp. Quality control was performed with agarose gel to ensure the correct major product, purify the DNA library using SPRI beads, quantify the concentration with a NanoDrop, and ensure equal pooling between samples for sequencing. The RNA (500 ng) of each sample that met the requirements of IR sequencing on concentration and purity was loaded into the cassette. Amplified libraries were multiplexed and pooled for sequencing on the Illumina NovaSeq platform with a 500-cycle kit and sequenced as 250 paired-end reads. The output of the immune receptor sequence covered within the first framework region through the beginning of the constant region including CDR2 and CDR3.

### 2.4 Data collection and bioinformatics analysis

The raw sequences obtained were collapsed by 10-bp UMI tags into the consensus FASTA format using MiGEC version 1.2.9 (https://migec.readthedocs.io/en/latest/) and were analyzed using the iRmap program (42, 43). Briefly, sequence reads were demultiplexed according to both Illumina dual indices incorporated during the amplification process and barcode sequences at the 5′ end of the reads from the constant region. For immune repertoire gene sequencing, paired-end fastqs were demultiplexed by 6-bp barcode using MiGEC version 1.2.9 and then stitched into a single read using pandaseq version 2.11. The merged reads were mapped using a Smith–Waterman algorithm to germline V, D, J, and C reference sequences using an IMGT reference library. To define the complementarity determining region 3 (CDR3), the position of CDR3 boundaries of reference sequences from the IMGT database was migrated onto reads through mapping results, and the resulting CDR3 regions were extracted and translated into amino acids. The dataset was condensed by the combination of UMIs and CDR3 regions to remove incorrect CDR3s introduced by sequencing and amplification. Reads with the same combination of CDR3 and UMI were condensed into one.

CSR rate:


$$CSR\;ratio=\frac{\sum^{}CSR\;num}{\sum_{i=0}^{n}copy_{i}}$$

Where CSR num is the accumulation of the number of copies of different immunoglobulins with the same type of CDR3. For example (below)

**Table:** 

CDR3	Region C	Copy
CARCYYGSGSWYFDLW	IGHM	2
CARCYYGSGSWYFDLW	IGHG12	3

For the sequence “CARCYYGSGSWYFDLW,” the CSR num is 5, and the bottom of the fraction shows the accumulation of the quantity of all copies.

SHM:

The number of mutations from CDR1 -> end of the V region, divided by the alignment length.

VJ usage:

This parameter is also a ratio and tells the type of usage frequency. For example, if the quantity of all copies is 100,000, IGHV1-1 will have 3 occurrences. So, IGHV1-1 usage is 3/100,000.

uCDR3 diversity: For calculating diversity with read, it is more accurate to use uCDR3 for the calculation.

Shannon entropy:


$$-\sum_{i=1}^{N}p_{i}log_{m}p_{i}$$

Where m , the base of the logarithm, determines the choice of units of the entropy measure. When a repertoire is composed of sequences evenly distributed, the Shannon entropy reaches its maximum, which is the logarithm of the number of unique sequences.

Gini:


$$\frac{\sum_{i=1}^{N}\sum_{j=1}^{N}|p_{i}-p_{j}|}{2N^{2}\bar{p}}$$

Where $p_{i}$ , $p_{j}$ refer to the frequency of the respective ith and *j*th sequences in the repertoire and $\bar{p}$ is the average of clone frequencies.

Gini is sometimes used to represent the clonal distribution of a repertoire. It is a measure of inequality that is widely used in economics to study wealth distribution.

D50:

Calculated as the percentage of clones that make up the top 50% of reads in the ranked clone distribution.

### 2.5 Statistical analysis

For continuous variables, data were presented as mean with standard deviation, and differences among groups were compared using Student’s *t*-test. Categorical variables were described as the number with percentage and compared using the chi-square test or Fisher’s exact test, as appropriate. All *p <*0.05 on two-sided tests was considered to be statistically significant. All statistical analyses were performed using the statistical software SPSS for Windows, Version 26.0 (IBM Corp., Armonk, NY, USA), GraphPad Prism V 8.0 (GraphPad Software, San Diego, California, USA), and R (ver4.2.0).

## 3 Results

### 3.1 Clinical demographics

This study enrolled 39 patients diagnosed with SARS-CoV-2 Omicron variant infection. They were divided into two groups: a mild disease group without pulmonary infiltration (non-PI, *n* = 21) and a severe/critical group with pulmonary infiltration (PI, *n* = 18). The PI group was significantly older than the non-PI group (74.33 ± 10.83 years vs. 62.10 ± 17.96 years, *p* = 0.016), had a lower lymphocyte count (1.05 ± 0.70 × 10^9^/L vs. 1.66 ± 0.72 × 10^9^/L, *p* = 0.003), a lower vaccination coverage rate (11.1% vs. 66.7%, *p* < 0.001), and a higher mortality rate (33.3% vs. 0%, *p* = 0.006) (**Table 1**). Additionally, there was no significant difference in underlying medical conditions (hypertension, diabetes mellitus, coronary heart disease, and cancer) between the two groups. Various studies (25–28) have reported lymphopenia in COVID-19 patients, characterized by a reduced number of lymphocytes and decreased expression of TCR and BCR. In this study, we also observed a significant reduction in lymphocyte count in PI patients compared to non-PI patients (**Table 1**).

**Table 1:** Baseline clinical characteristics of the two groups of patients.

	Non-PI group (*n* = 21)	PI group (*n* = 18)	*p*
Age (mean/SD, years)	62.10 (± 17.96)	74.33 (± 10.83)	0.016
Gender/F, *n* (%)	13 (61.9)	6 (33.3)	0.075
Inactivated SARS-CoV-2 vaccine, *n* (%)	14 (66.7)	2 (11.1)	0.001
1 dose, *n* (%)	1 (4.8)	0 (0)	
2 doses, *n* (%)	4 (19.0)	2 (11.1)	
3 doses, *n* (%)	9 (42.9)	0 (0)	
Hypertension, *n* (%)	11 (52.4)	12 (66.7)	0.366
Diabetes mellitus, *n* (%)	6 (28.6)	5 (26.3)	0.956
Coronary heart disease, *n* (%)	3 (14.3)	0 (0)	0.235
Malignancy, *n* (%)	0 (0)	3 (16.7)	0.089
Lymphocyte (10^9^/L)^a^	1.66 (± 0.72)	1.05 (± 0.70)	0.003
Baseline value of CT^b^	27.81 (± 4.63)	28.44 (± 4.36)	0.562
Death	0 (0)	6 (33.3)	0.006
Sampling time from diagnosis (days)	11.10 (± 2.89)	11.94 (± 2.07)	0.316

a The normal range of lymphocytes is 1.1–3.2 × 10^9^/L.

b The value of CT higher than 35 was considered a negative result.

### 3.2 Comprehensive immune repertoire differences between PI and non-PI patients

**Figure 1:**
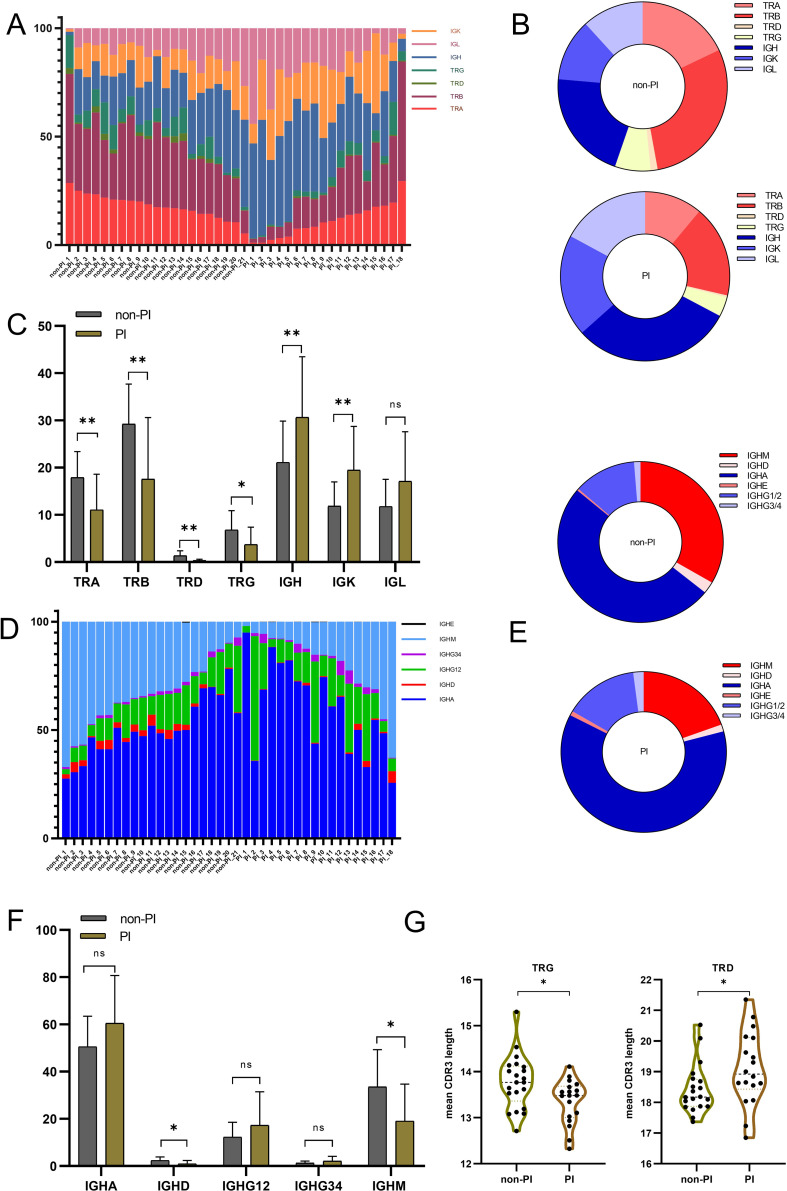
The overall TCR and BCR immune repertoire of patients with SARS−CoV−2 Omicron infection without or with pulmonary infiltration were sequenced, and the reads of each gene were aligned and counted. Data were collected from 39 biological replicated experiments: *n* = 21 without pulmonary infiltration (non-PI) and *n* = 18 with pulmonary infiltration (PI). **(A)** Percentage of the seven-chain repertoire for patients with SARS−CoV−2 Omicron infection without or with pulmonary infiltration. Each bar represents the composition ratio in one sample. **(B)** The composition ratio of the seven-chain immune repertoire in the two groups by counting unique CDR3. **(C)** The percentage [details from **(A)**] for each TCR and BCR chain between the two groups. **(D)** Comparison of each Ig isotype percentage for patients with SARS−CoV−2 Omicron infection without or with pulmonary infiltration. **(E)** The composition ratio of Ig isotypes in the two groups by counting unique CDR3. **(F)** The percentage [details from **(D)**] for each BCR isotype between the two groups. **(G)** Mean CDR3 length of the TRG and TRD chains between the two groups. * means P≤ 0.05, ** means P≤ 0.01, ns means No significance.

Previous studies have shown characteristic alterations in the TCR/BCR immune repertoire following SARS-CoV-2 infection (18, 29). However, only a few immune repertoire profiling studies for the SARS-CoV-2 Omicron variant infection have been reported. We collected peripheral blood samples from all 39 patients and amplified the immune repertoire, including all TCR (TRA, TRB, TRD, TRG) and BCR (IGH with its isotypes IGK and IGL), in a single, unbiased PCR reaction. Several studies have revealed the activation patterns and dynamics of the adaptive immune cell response to SARS-CoV-2 infection (20, 30). We observed a pattern of increased IGH and IGK chains and decreased TRA, TRB, TRD, and TRG chains within the total immune repertoire in PI patients compared to non-PI patients (**Figures 1A–C**), indicating a hyperactivation of humoral immunity over cellular immunity in patients with severe to critical symptoms. We further analyzed the B-cell repertoire composition by calculating unique CDR3 sequences. IGHA was predominant in the BCR repertoire of all patients, while the proportions of IGHD and IGHM were significantly lower in PI patients compared to non-PI patients (**Figures 1D–F**). Previous studies have reported a dominant IGHA response to viral infections, including SARS-CoV-2 (19, 31), consistent with our findings. Additionally, reduced IGHD and IGHM in PI patients suggest that IGHD and IGHM may offer protection against SARS-CoV-2 Omicron variant lung tissue invasion. Lastly, we measured the mean CDR3 length across all seven immune repertoire chains. The TRD and TRG chains in PI patients had longer and shorter CDR3 lengths, respectively, compared to non-PI patients (**Figure 1G**), while the other chains showed no significant differences between the groups (**Supplementary Figures 1A–E**).

In summary, patients with pulmonary infiltration exhibited a distinct immune repertoire, characterized by decreased TCR and increased BCR expression. Specifically, IGHD and IGHM showed reduced expression, and the TRD/TRG chains had longer/shorter CDR3 lengths in PI patients, potentially serving as an immune repertoire signature to distinguish patients with or without pulmonary infiltration.

### 3.3 Altered adaptome clonality and diversity between PI and non-PI patients

**Figure 2:**
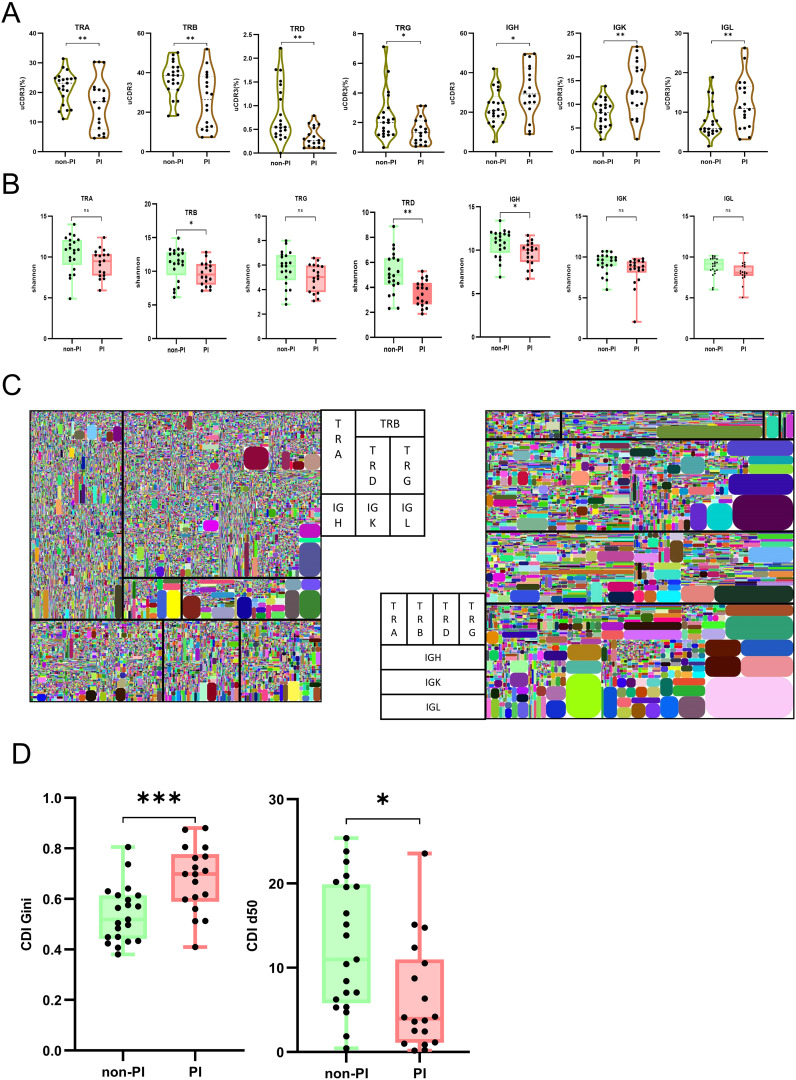
TCR and BCR abundance, clonal expansion, and diversity analysis between patients with SARS−CoV−2 Omicron infection without or with pulmonary infiltration. **(A)** The abundance of each chain for immune repertoire adaptome is measured by the number of unique CDR3 (uCDR3); the uCDR3 counts of each chain are presented for the two groups as mean value ± SD in the boxplots. **(B)** The diversity was demonstrated by the Shannon index at the level of unique uCDR3 clones, as shown in TRA, TRB, TRD, TRG, IGH, IGK, and IGL. **(C)** Seven-chain repertoire tree maps for two representative subjects from each group. Each square represents a chain, with its size being representative of its relative expression out of the seven chains. All tree maps should be read from left to right and then from top to bottom in the following order: TRA, TRB, TRD, TRG, IGH, IGK, and IGL. Each rounded rectangle color block is a clone. **(D)** The diversity was demonstrated by CDI d50 and CDI Gini. * means P≤ 0.05, ** means P≤ 0.01, *** means P≤ 0.001, ns means No significance.

A unique CDR3 (uCDR3) sequence, also known as a clonotype, targets specific antigens, thus reflecting the overall adaptive immunity. PI patients showed a reduction in uCDR3 for TRA, TRB, TRD, and TRG, but an increase in uCDR3 for IGH, IGK, and IGL, compared to non-PI patients (**Figure 2A**). Numerous studies have highlighted a correlation between reduced immune repertoire diversity, an impaired adaptive immune system, and various diseases (28). To determine if PI patients had a more severely damaged repertoire than non-PI patients, we assessed the diversity of the TCR and BCR repertoires in all samples. Diversity, quantified by Shannon entropy, indicates the uniformity of the immune repertoire. PI patients exhibited a significant reduction in Shannon entropy for three immune repertoire chains, TRB, TRD, and IGH, compared to non-PI patients (**Figures 2B, C**). The clonal diversification index (CDI) analysis was used to assess population unevenness, mitigating potential biases from varying RNA molecule counts per cell. PI patients exhibited a reduced CDI d50 and an increased CDI Gini compared to non-PI patients (**Figure 2D**). In summary, our findings reveal diminished TCR/BCR clonality and diversity in PI patients, suggesting a further deteriorated adaptome.

### 3.4 Characteristic TRBV/J and IGHV/J gene usage and shared CDR3 sequences between PI and non-PI patients

**Figure 3:**
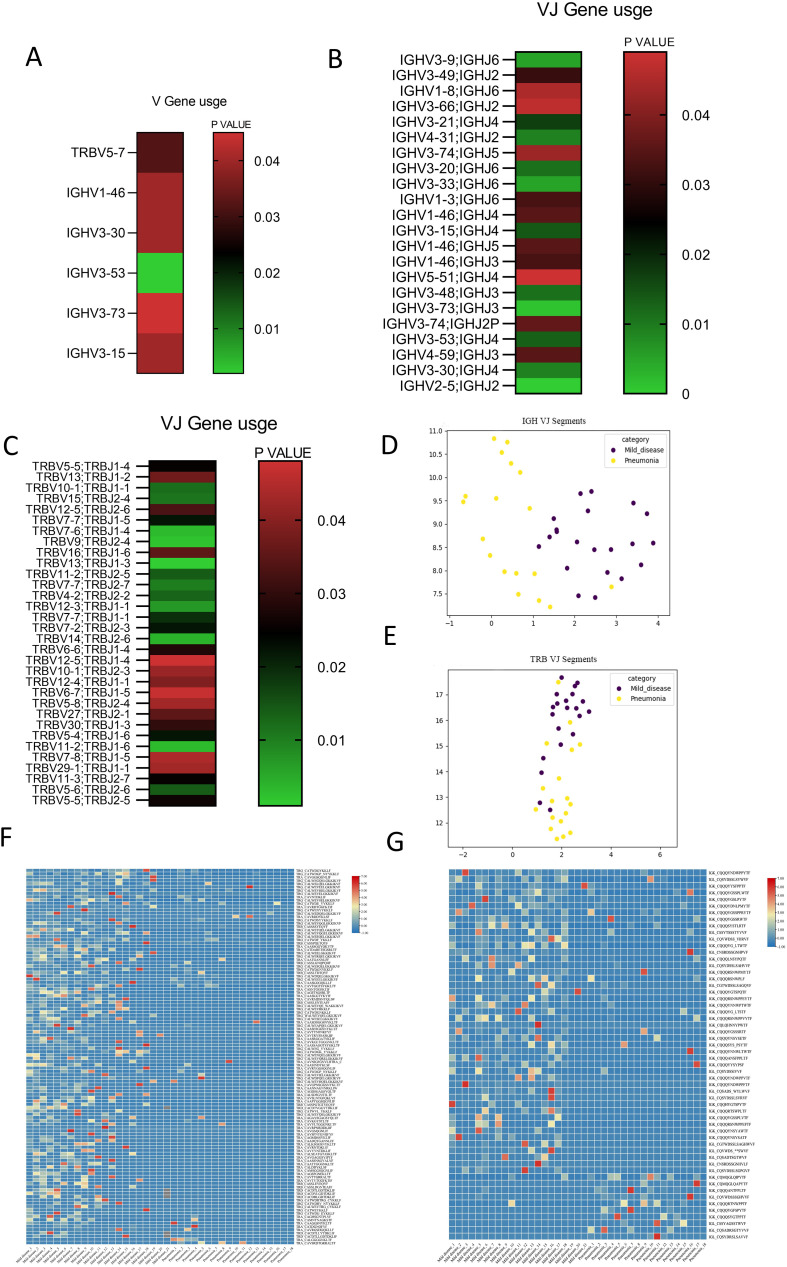
Distinct V/J preference usage and share of CDR3 clones between patients with SARS−CoV−2 Omicron infection without or with pulmonary infiltration. Cluster analysis heat map of the expression level of genes based on the **(A)** difference of the IGHV gene and the TRBV gene between the two groups. Heat maps of TRBV–TRBJ **(B)** and IGHV–IGHJ conjunctions **(C)** between the two groups are presented, showing preferred TRBV–TRBJ and IGHV–IGHJ pairs. Uniform manifold approximation and projection (UMAP) based on the abundance of TRBV **(E)** and IGHV **(D)** segments; the distance between the dots indicates the degree of dissimilarity between samples. Cluster analysis heat map for CDR3 between patients with SARS−CoV−2 Omicron infection without or with pulmonary infiltration for TCR **(F)** and BCR **(G)**.

The TRBV and IGHV families encode the variable segments of the TRB and IGH chains, significantly impacting TCR/BCR diversity (13, 30). We calculated the frequencies of TRBV, TRBJ, IGHV, and IGHJ genes in PI patients and compared them with those in non-PI patients. Siginificant different usage of TRBV5-7, IGHV1-46, IGHV3-30, IGHV3-15, IGHV3-53, and IGHV3-73 was found in PI patients compared to non-PI patients (**Figure 3A**). Furthermore, significant differences in TRBV_TRBJ and IGHV_IGHJ pairs were observed between the two groups of patients (**Figures 3B, C**). Uniform manifold approximation and projection (UMAP) analyses were conducted based on the abundance of TRBV (**Figure 3B**) and IGHV (**Figure 3C**) segments. Significant differences between PI and non-PI patients were noted, with distinct separation of TRBVJ and IGHVJ clusters (**Figures 3D, E**). To assess the prevalence of shared CDR3 sequences among the two groups, we combined all sequencing data for analysis. Shared CDR3 sequences across seven chains were expressed at different levels between PI and non-PI patients. The expression levels of uCDR3 and representative sequences were plotted, revealing significant differences between PI and non-PI patients (*p* < 0.05) (**Figures 3F, G**). Each column represents a patient, and each cell in a row indicates the expression frequency of a CDR3 sequence, with the color indicating the expression level (blue for low expression and red for high expression). Among the seven chains, shared CDR3 sequences were found in TRA, TRB, TRD, TRG, IGL, and IGK, but not in IGH (**Figure 3G**), likely due to the high frequency of SHM in the IGH chain. Between PI and non-PI patients, numerous CDR3 sequences showed differential expression, with the most highly expressed shared CDR3 sequences found in non-PI patients (**Figures 3F, G**), suggesting that these CDR3 sequences may offer protection against pulmonary infiltration. Meanwhile, a small subset of shared CDR3 sequences in the IGK and IGL chains was preferentially expressed in the PI group but absent in the non-PI group, indicating that these clones may be associated with the progression of the infection. In summary, we identified significant differences in V and V-J gene usage between PI and non-PI patients, along with a unique CDR3 signature that was differentially expressed between the two groups. These molecular markers could be further investigated for their roles in the etiology and progression of COVID-19 pulmonary infiltration and their potential as diagnostic biomarkers.

### 3.5 Elevated CSR in PI patients compared to non-PI patients

**Figure 4:**
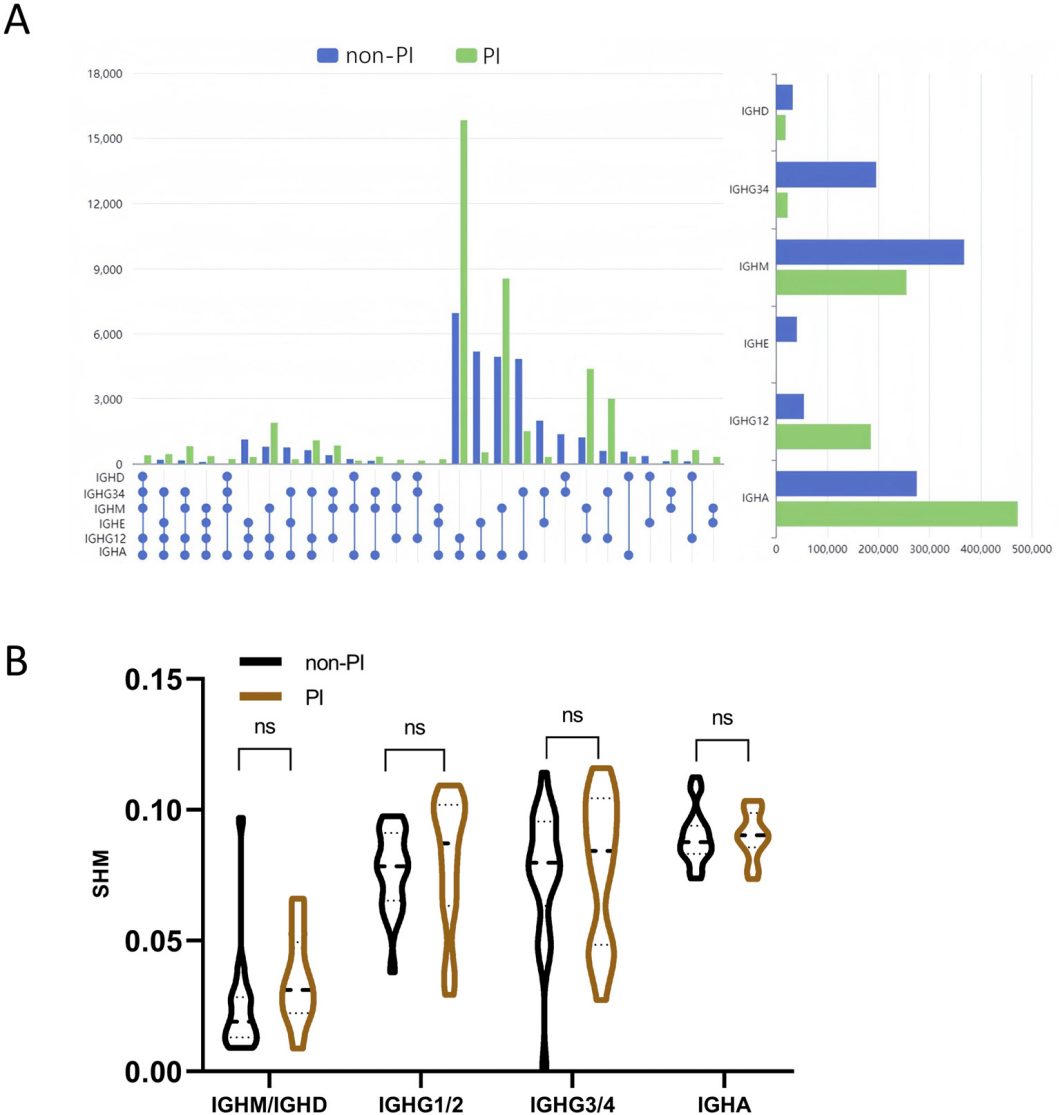
Analysis of class switch recombination (CSR) and somatic hypermutation (SHM) for BCR. **(A)** The CSR of the six Igs (IGHA, IGHD, IGHE, IGHG12, IGHG34, and IGHM) between the two groups. The spheres represent different immunoglobulin isotypes, and the line in between the dots indicates class switch between these two isotypes. Isotype switch with a significant difference between the two groups (*p*-value < 0.05) is presented. **(B)** Somatic hypermutation ratio in the four subtypes of Ig (IGHM and IGHD are shown in the graph as a whole). ns means No significance.

The B-cell repertoire is characterized by unique events of CSR and SHM (32, 33). Considering the six main isotypes (IGHD, IGHM, IGHG1/2, IGHG3/4, IGHE, and IGHA), we used the percentage of unique CDR3 sequences observed in more than one isotype to evaluate class switching among multiple isotypes. We observed dramatic differences in CSR rates among different isotypes between PI and non-PI patients (**Figure 4A**). The CSR rates for IGHE–IGHG12–IGHA, IGHG34–IGHE–IGHA, IGHD–IGHM–IGHA, IGHE–IGHA, IGHG34–IGHA, IGHG34–IGHE, IGHD–IGHA, and IGHD–IGHE were significantly higher in non-PI patients, while the remaining isotypes had higher CSR rates in the PI group. In PI patients, the IGHA isotypes of BCR most frequently undergo class switching. Overall, the cumulative CSR rates in non-PI patients were higher for IGHD, IGHG34, IGHM, and IGHE and lower for IGHG12 and IGHA (**Figure 4A**). Previous studies have reported changes in SHM among COVID-19 patients (20). In our study, no significant differences in SHM were observed for IGM/IGD, IGHG1/2, IGHG3/4, and IGHA between the two groups (**Figure 4B**). In summary, PI patients exhibited distinct B-cell repertoire patterns, characterized by lower CSR rates in IGHD, IGHG34, IGHM, and IGHE and higher CSR rates in IGHG12 and IGHA.

### 3.6 The inactivated COVID-19 vaccine triggered a specific adaptome response and protected patients from severe or critical conditions

**Figure 5:**
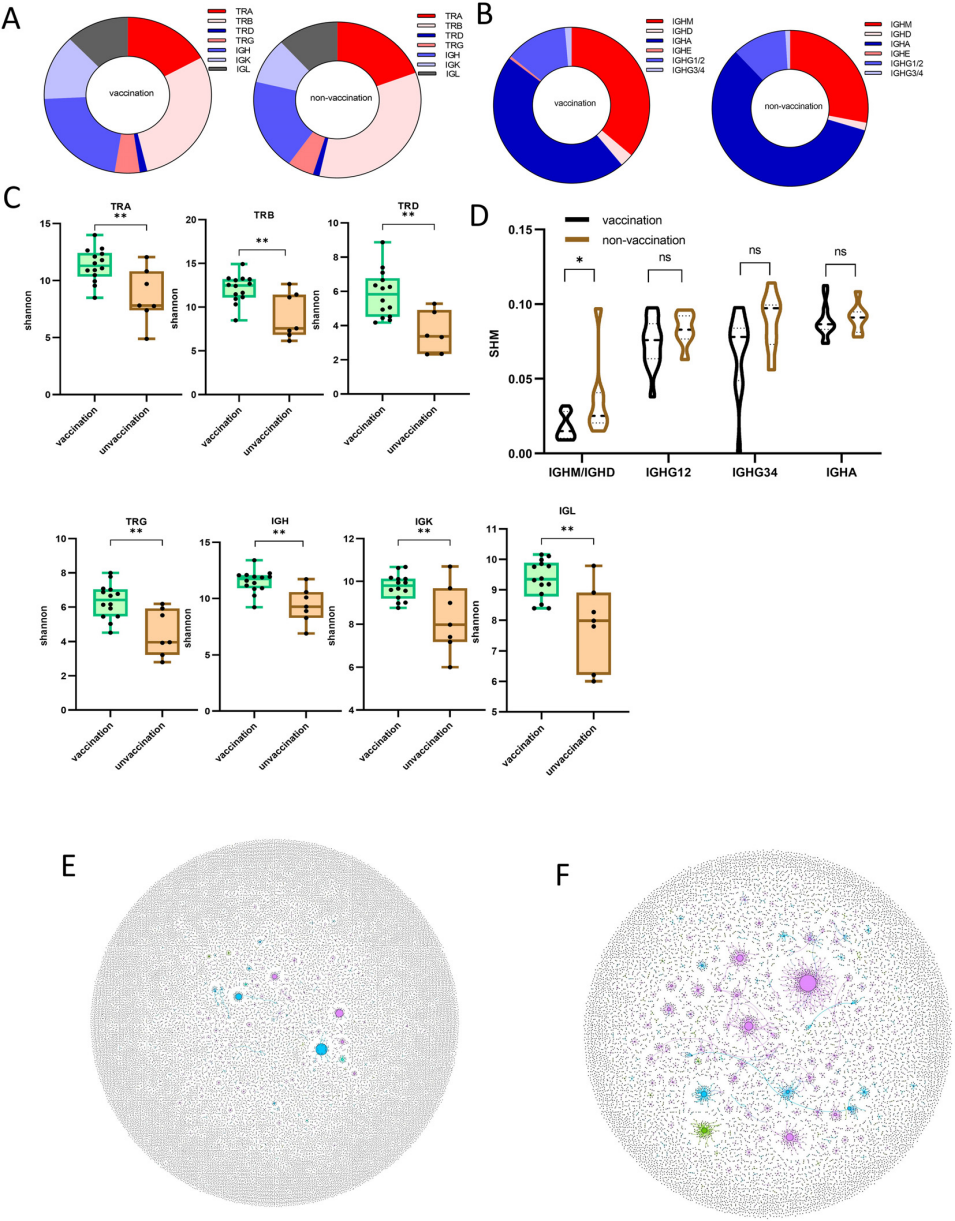
Immune repertoire pattern difference between vaccinated and unvaccinated patients after being infected with SARS−CoV−2 Omicron. **(A)** The composition ratio of the seven-chain immune repertoire in vaccinated and unvaccinated patients. **(B)** Comparison of each Ig isotype percentage for patients with SARS−CoV−2 Omicron infection with or without prior vaccination. **(C)** The diversity was demonstrated by the Shannon index at the level of unique uCDR3 clones, as shown in TRA, TRB, TCD, TRG, IGH, IGK, and IGL for patients with SARS−CoV−2 Omicron infection with or without prior vaccination. **(D)** Somatic hypermutation ratio in the four subtypes of Ig (IGHM and IGHD are shown in the graph as a whole) of patients with SARS−CoV−2 Omicron infection with or without prior vaccination. **(E)** B-cell hypermutation is shown in two representative individual samples **(F)**, in which each dot represents a unique B-cell uCDR3. The size of each dot denotes the relative frequency of that uCDR3 clone, and the dots connected by a line represent hypermutation. * means P≤ 0.05, ** means P≤ 0.01, ns means No significance.

The inactivated vaccine has been proven to be an effective strategy for preventing the COVID-19 pandemic (34). However, the rapid evolution of the virus has led to numerous variants and mutations, compromising the efficacy of the original vaccines. Since November 2021, the SARS-CoV-2 Omicron variant has become the most dominant strain globally, as also found in the sample cohort of this study. In this study, of the 21 patients with mild cases, 14 were vaccinated, whereas only 2 of the 18 severe/critical cases had been vaccinated. This validates that the inactivated vaccine provides significant protection against severe or critical conditions in patients with SARS-CoV-2 Omicron infection (**Table 1**). To further explore the adaptome features of vaccinated patients, we compared the immune repertoire features in mild cases with and without prior vaccination. The overall composition of the seven-chain immune repertoire and the proportion of B-cell isotypes showed no difference between vaccinated and unvaccinated subjects (**Figures 5A, B**). Interestingly, the diversity of all TRA, TRB, TRD, TRG, IGH, IGK, and IGL chains, as measured by the Shannon index, was significantly lower in unvaccinated compared to vaccinated patients (**Figures 5C**), indicating more severe damage to the immune system of unvaccinated patients upon infection. Finally, we observed a higher SHM rate in non-vaccinated compared to vaccinated patients (**Figures 5D–F**). The SHM rate has been reported to be related to the efficacy of vaccination (35), reflecting the preparedness of B cells against infection. Therefore, the inactivated COVID-19 vaccine triggered a lesser B-cell IGHM/IGHD SHM response upon SARS-CoV-2 Omicron infection, preserved the diversity of the entire adaptome immune repertoire, and provided clinical protection against severe or critical conditions caused by SARS-CoV-2 Omicron infection.

## 4 Discussion

Since December 2019, the COVID-19 pandemic has had a devastating impact on the global economy and health. Furthermore, SARS-CoV-2 continues to mutate, generating new variants, with Omicron becoming the dominant variant so far (36, 37). The host’s immune response against SARS-CoV-2 infection not only plays an antiviral role but also leads to simultaneous pathogenic injury of the organs and tissues, especially in the lungs of patients with COVID-19, which determines the disease severity and outcome (38, 39). Therefore, it is of great importance to understand immune responses to SARS-CoV-2 infection, especially the SARS-CoV-2 Omicron variant. To address this, we profiled the immune repertoire landscape, characterizing the composition and diversity using iR-RepSeq-plus 7-Chain Cassette in patients with SARS-CoV-2 Omicron infection, both with and without pulmonary infiltration (PI and non-PI patients).

This study delineated the disrupted immune repertoire patterns in patients with SARS-CoV-2 Omicron infection, particularly those with PI. Our results revealed reduced diversity in TRB, TRD, and IGH chains in PI patients compared to non-PI patients, potentially constituting a risk factor for disease progression. Several studies found that SARS-CoV-2 infection caused the TRB chain or IGH chain diversity to decline (16, 29, 30). By definition, an exaggerated focus would limit immune cell diversity that may be particularly important antibody-escape variants of concern and/or other coinfections. We also observed preferential V(D)J gene segment usage of IGH and TRB between the two groups of patients. The skewed usage of the V/J segments may be associated with immune dysfunction and the pathogenesis of the disease. Upon infection, SARS-CoV-2 antigens can lead to the targeted rearrangement and excessive abnormal cloning of one or a few V subfamilies, and the cloning of other immune cells may be suppressed by the dominant immune cell clones, which may result in impaired immune function and decreased ability to clear the virus (30).

The distribution of CDR3 sequence lengths is another key feature that provides an integrative view of repertoire composition. Biases in CDR3 length are often observed in epitope-specific repertoire (40). In our study, we observed differences in the mean CDR3 length of the TRD and TRG chains between the two groups. We found a cluster of shared TCR and BCR CDR3 clones, most of which were highly shared among non-PI patients. Therefore, these TCR and BCR clonotypes shared between individuals are likely stimulated against common antigens and are thought to play a significant role in the efficacy of pathogen-specific responses and the control of infection. Thus, if linked to certain infections, such TCRs or BCRs could become invaluable tools for immune diagnosis of human disease and vaccine development. Furthermore, in our cohort, IGHA presented the highest proportion in total BCR repertoire in the two groups. Meanwhile, compared with the other isotypes, the IGHA isotypes of BCR frequently undergo most class switches in PI patients. This discovery thus highlights the importance of developing and using inhaled vaccine. Meanwhile, for SARS-CoV-2, neutralizing antibodies primarily target five structural regions of the spike protein, especially the receptor-binding domain (RBD), and can block the virus from infecting host cells. Previous studies have shown that most neutralizing antibodies in certain populations are encoded by the IGHV1-2 gene family. However, our research found an increased usage of IGHV3 in pneumonia patients. Additionally, human neutralizing antibodies against COVID-19 are predominantly of the IgA class, and our results indicate a higher rate of CSR to IGHA in pneumonia patients. This may suggest increased production of non-neutralizing antibodies, potentially leading to immune-related lung damage.

The above results indicated that a disruptive immune repertoire may contribute to COVID-19 progress. Vaccine remains the most effective approach against the COVID-19 global pandemic. Meanwhile, we found a higher vaccination coverage in the mild disease group. By immune repertoire sequencing, compared to unvaccinated patients, upon SARS-CoV-2 infection, vaccinated patients possess increased IGH chain and TRB chain diversity, and more IGHM and IGHD SHMs were observed in unvaccinated patients. To some extent, vaccination increases the immune system’s buffering capacity to ameliorate the SARS-CoV-2 Omicron infection. A previous study (35) had a different observation, and we believe the contradictory conclusion is due to the different mechanisms of the vaccine. Their study focused on mRNA vaccines, targeting specific immunity against parts of the S protein, while our study encompassed subjects vaccinated with the inactivated vaccine, eliciting a broader immune response due to a wider range of epitopes. Our findings still have shortcomings, including the lack of strict negative controls (patients with no infection) and a relatively small number of cases included, which may lead to instability in the results. However, our findings still aid in understanding the immune repertoire in patients infected with the SARS-CoV-2 Omicron variant.

In summary, this study provides a comprehensive overview of the immune repertoire in patients infected with the SARS-CoV-2 Omicron variant. We also found a correlation between the impaired immune repertoire and the clinical severity of COVID-19. Finally, our study suggests that the TCR/BCR repertoire could serve as a biomarker for predicting COVID-19 severity, offering diagnostic value and guiding more precise therapeutic strategies.

## Data Availability

The data presented in the study are deposited in the National Genomics Data Center repository, accession number HRA009242.

## Supplementary material

Supplementary Figure 1
**(A-E)** Mean CDR3 length of TRA, TRB, IGK, IGH and IGL chains between two groups.
