# Supplementary figures and images for: A comprehensive immune repertoire signature distinguishes pulmonary infiltration in SARS-CoV-2 Omicron variant infection
Source: Front Immunol. 2024 Dec 17;15:1486352. doi: 10.3389/fimmu.2024.1486352 (PMC11685115; doi:10.3389/fimmu.2024.1486352)

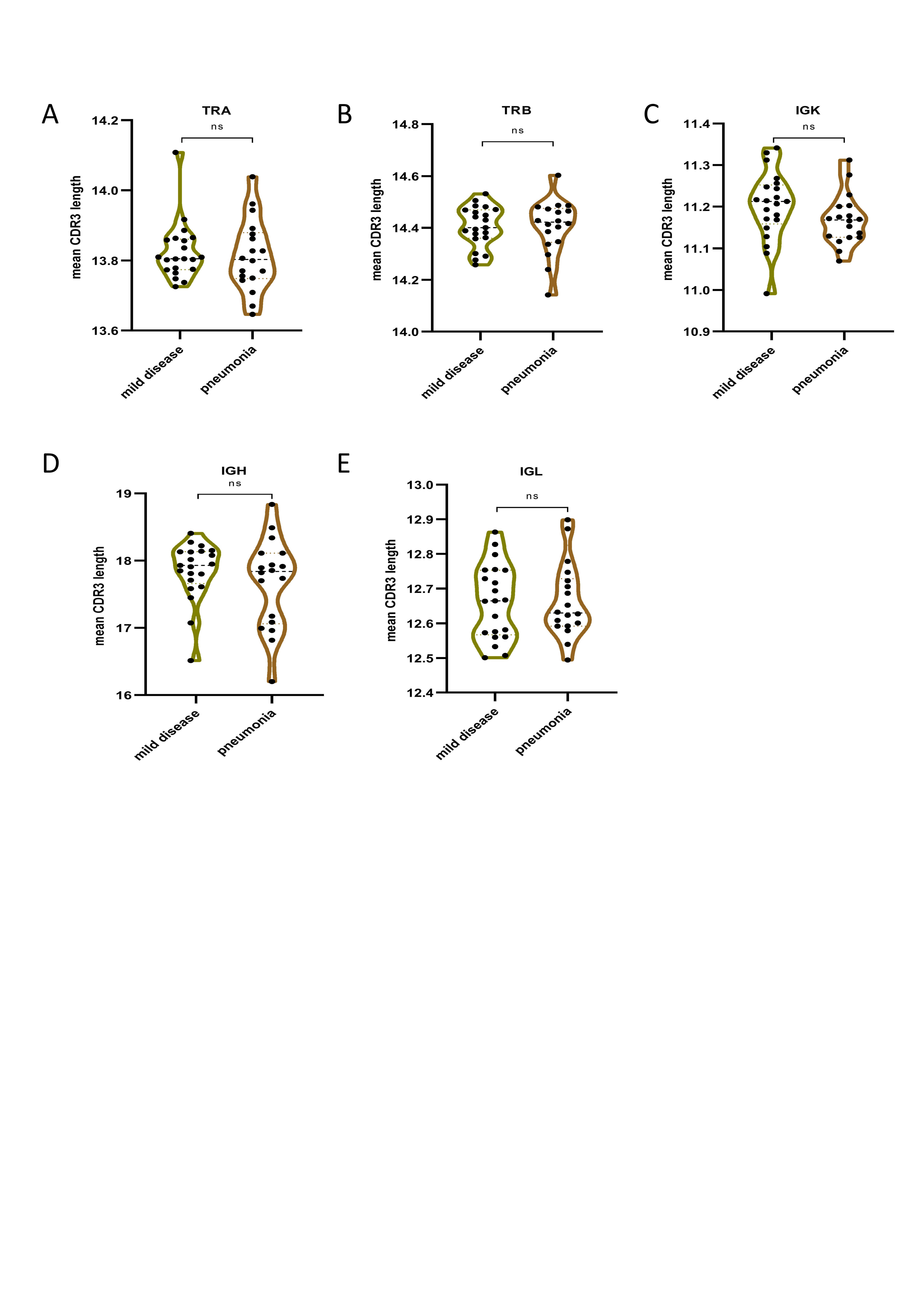

Supplement: Supplementary Figure 1 — (A-E) Mean CDR3 length of TRA, TRB, IGK, IGH and IGL chains between two groups. [file Image1.jpeg]
